# Adding Two Antimicrobial Glasses to an Endodontic Sealer to Prevent Bacterial Root Canal Reinfection: An In Vivo Pilot Study in Dogs

**DOI:** 10.3390/antibiotics10101183

**Published:** 2021-09-28

**Authors:** Álvaro Zubizarreta-Macho, Cristina Rico-Romano, María Jesús Fernández-Aceñero, Jesús Mena-Álvarez, Belén Cabal, Luis Antonio Díaz, Ramón Torrecillas, José Serafín Moya, Roberto López-Píriz

**Affiliations:** 1 Department of Endodontics, Faculty of Health Sciences, Alfonso X el Sabio University, 28691 Madrid, Spain; azubimac@hotmail.com (Á.Z.-M.); cromaric@uax.es (C.R.-R.); jmenaalvarez@gmail.com (J.M.-Á.); 2 Department of Surgery, Faculty of Medicine and Dentistry, University of Salamanca, 37008 Salamanca, Spain; 3 Pathological Anatomy Service, San Carlos Clinical Hospital, 28040 Madrid, Spain; mgg10167@gmail.com; 4 Nanomaterials and Nanotechnology Research Centre (CINN), Consejo Superior de Investigaciones Científicas (CSIC), Universidad de Oviedo (UO), Principado de Asturias (PA), Avenida de la Vega 4-6, 33940 El Entrego, Spain; la.diaz@cinn.es (L.A.D.); r.torrecillas@cinn.es (R.T.); jsmoya@icmm.csic.es (J.S.M.); 5 Advanced Oral Surgery Institute (ICOA), 28691 Madrid, Spain

**Keywords:** endodontics, root canal sealer, bioactive glass, bactericidal, *Enterococcus faecalis*, bactericidal effect, apical periodontitis

## Abstract

Current endodontic procedures continue to be unsuccessful for completely removing pathogens present inside the root canal system, which can lead to recurrent infections. In this study, we aimed to assess the antimicrobial capacity and tissue response of two inorganic bactericidal additives incorporated into a paste root canal sealer on contaminated root dentin in vivo. An experimental study was performed in 30 teeth of five Beagle dogs. After inducing microbiological contamination, root canal systems were treated by randomly incorporating one of two antimicrobial additives into a commercial epoxy-amine resin sealer (AH Plus), i.e., G3T glass-ceramic (n = 10) and ZnO-enriched glass (n = 10); 10 samples were randomized as a control group. After having sacrificed the animals, microbiological, radiological, and histological analyses were performed, which were complemented with an in vitro bactericidal test and characterization by field emission scanning electron microscopy. The tested groups demonstrated a non-significant microbiological reduction in the postmortem periapical index values between the control group and the bactericidal glass-ceramic group (*p* = 0.885), and between the control group and the ZnO-enriched glass group (*p* = 0.169). The histological results showed low values of inflammatory infiltrate, and a healing pattern characterized by fibrosis in 44.4% of the G3T glass-ceramic and 60.0% of ZnO-enriched glass. Bactericidal glassy additives incorporated in this root canal sealer are safe and effective in bacterial reduction.

## 1. Introduction

Bacterial infection plays a key role in the establishment and development of necrotic processes of dental pulp and the formation of periapical lesions [1]. Chronic apical periodontitis is described as a dental disease accompanied by periapical tissues pathological changes. It is caused by pulp necrosis and characterized by a high level of bacterial contamination along the root canal system [2].

Elimination of the pathogenic microflora from the root canal system is an important factor in the prognosis of endodontic therapy. Negative microbiological cultures obtained from the root canal system show an endodontic success rate close to 94%. However, positive microbiological cultures reduce endodontic success rate to 68% [3]. More than 20 million root canals are performed annually in the United States [4], of which 22.8% develop an apical periodontitis, and 10% require a root canal retreatment [5], which has demonstrated a lower success rate than a conventional endodontic treatment (80%) [6]. In most of the cases, the etiology of endodontic failure is related to persistent or secondary endodontic infection [7], frequently associated with facultative anaerobic Gram-positive microorganisms. Among these, especially *Enterococcus faecalis* (*E. faecalis*) has been proven to be highly resistant to conventional antimicrobial agents and to be able to colonize dentinal tubules despite endodontic treatment; therefore, it is able to reinfect the root canal system and subsequently lead to endodontic treatment failure [1,8].

Current endodontic procedures continue to be unsuccessful for completely removing pathogens present inside the root canal system. Therefore, it is important to consider a novel approach that indefinitely maintains an antimicrobial effect over the residual bacterial load present in the root canal system to avoid recurrent apical periodontitis. Conventional endodontic sealers have demonstrated limited antibacterial effectiveness over time, mediated by released substances during setting time. Among the root canal sealers, epoxy resin-based sealers present short-term antibacterial effects, due to the formaldehyde or bisphenol A diglycidyl ether released during their setting process [9]. Likewise, zinc oxide eugenol-based sealers release eugenol particles during the curing stage [10,11], whereas silicon-based sealers and calcium hydroxide-based sealers report a weak antibacterial effect [12]. However, calcium silicate- and phosphate-based bioceramic sealers and MTA-based sealers owe their action to their high pH and the materials and ions released during setting [11,13].

Over the past years, attempts have been made to modify root canal sealers with antibacterial additives such as quaternary ammonium compounds, chlorhexidine, iodoform, natural extracts, antibiotics, antifungal drugs, antimicrobial agent-functionalized nanoparticles, and calcium hydroxide [14]. Copper–calcium hydroxide (Cupral) is another type of antimicrobial additive that has shown promising effects in the treatment of apical periodontitis, in vitro against endodontic pathogens [15] and also clinically [16]. The use of different methodologies for evaluating the antimicrobial efficacy of these additives makes direct, vis-à-vis comparisons impossible. Antimicrobial additives exert their effects based on one of three different strategies: (1) through their local release, (2) through a contact-killing strategy, or (3) through a multi-functional strategy to produce synergetic properties [17]. Each methodology has inherent advantages, but also disadvantages such as low efficiency, alteration of the physical properties of the sealer, or even toxicity. In any case, further studies are still required to better comprehend their clinical impact.

Recently, a new generation of inorganic antimicrobial materials based on zinc oxide (ZnO)-enriched glass, have evidenced long-term bactericidal activity (mean log microbial reduction >3) [18]. Furthermore, a new glass-ceramic (G3T), composed of combeite and nepheline in a residual soda-lime glassy matrix, has demonstrated the capacity to inhibit bacterial biofilm adhesion and formation [19]. Long-term antimicrobial properties of these novel materials, incorporated as additives into endodontic sealers, could possibly prevent root canal bacterial reinfection. In this study, the effect of two antimicrobial glass/glass-ceramic particulate filler additives, added to one of the most common endodontic sealers (i.e., AH Plus) used in the conventional nonsurgical root canal treatment to avoid apical periodontitis, is evaluated through an in vitro and an in vivo pilot study with Beagle dogs.

## 2. Results

### 2.1. In Vitro Efficacy of the Glass and Glass-Ceramic Additives against E. faecalis

In vitro studies compared the antimicrobial activity of a commercially available root canal sealer (i.e., AH Plus) filled with three different antimicrobial glasses (i.e., G3T glass-ceramic, ZnO-enriched glass, and 45S5 Bioglass^®^ (Novabone Products LLC, Alachua, FL, USA).

**Figure 1:**
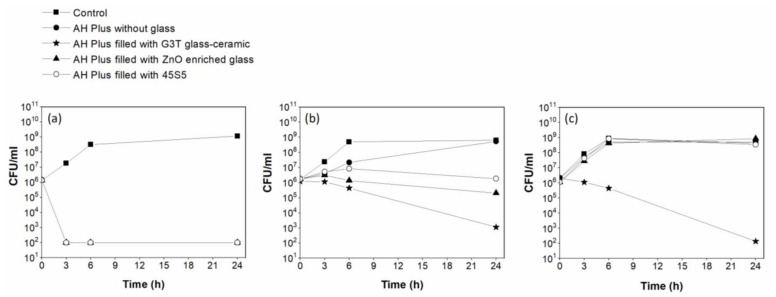
Antibacterial activity of glass-filled AH Plus: (**a**) Samples were used within 20 min after mixing; (**b**) samples were allowed to set for 48 h in a humid atmosphere at 37 °C; (**c**) samples were allowed to set for 48 h in a humid atmosphere at 37 °C, and then aged for 5 days in phosphate buffered saline (PBS) at 37 °C. All the assays were conducted in triplicate, each point on the curve is the average of three measures.

The glass-filled sealers were evaluated in this study by using the direct contact test, and showed different inhibitory effects depending on the time interval between mixing and testing (Figure 1). The AH Plus root canal sealers that contained different antimicrobial glasses were equally effective in inhibiting bacterial growth (*p* < 0.05) and exerted bactericidal effect until 20 min after mixing (Figure 1a). After 48 h of mixing, the antibacterial activity of AH Plus was lost (Figure 1b), whereas for the antimicrobial glass-filled sealers, it was maintained although to a lesser extent than the 20-minute samples. After 7 days from mixing, only AH Plus with 15 wt% of the antimicrobial G3T glass-ceramic still exerted antibacterial activity (*p* < 0.05). No antibacterial activity was found for the other glasses (Figure 1c).

### 2.2. In Vivo Evaluation of the Glass and Glass-Ceramic Additives

During the root canal treatment, one root canal instrument was separated into the root canal system (positive control group) and one tooth was fractured during extraction procedures (G3T glass-ceramic study group), and therefore these excluded from the study.

#### 2.2.1. Histopathological Results

The histological parameters that were selected for this study all related to the quality of the healing process and the presence of inflammatory response that could delay healing. All these parameters were evaluated, attending to semiquantitative histomorphometric criteria which included:

- Newly formed periapical bone tissue (presence or absence);
- Vascularization, i.e., presence or absence of neovessels, as shown by the lack of a well-developed wall structure;
- Presence of necrotic periapical bone tissue (presence or absence);
- Presence and intensity periapical inflammatory infiltrate, considered to be mild (<10), moderate (11–20) or severe (>20) according to the number of inflammatory cells per high power field (HPF, x40);
- Degree and maturity of fibrosis, measured as the percentage of the HPF corresponding to fibrosis (stained green with trichrome stain) and the percentage of cells in fibrosis (young fibrosis is moderately cellular as opposed to scars, which are acellular or paucicellular);
- Type of cells predominant in the inflammatory response, i.e., lymphocytes, macrophages or polymorphonuclear cells.

##### Inflammatory Infiltrate

**Figure 2:**
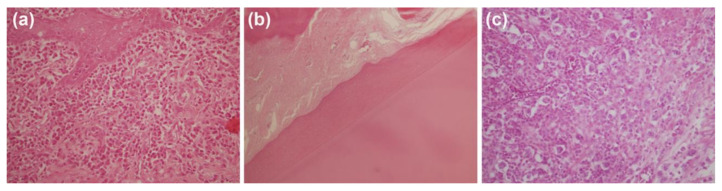
Representative picture of hematoxylin and eosin staining of (**a**) Granulation-like inflammation (×400); (**b**) mature fibrosis (×400); and (**c**) inflammatory abscess (×200), developed in the periapical region of samples sealed with ZnO-enriched glass, G3T glass-ceramic, and the control group, respectively.

The healing pattern of periapical tissues developed low values of inflammatory infiltrate; nevertheless, mild inflammation was present in 11.1% of the teeth treated with G3T glass-ceramic and 30.0% of the teeth treated with ZnO-enriched glass (Figure 2a). Figure 2b shows dispersed moderate inflammation. Lymphocytes were present in 82.4% of the teeth treated with G3T glass-ceramic and 79% of the teeth treated with ZnO-enriched glass. Nevertheless, 10.6% and 15.7% of the teeth treated with G3T glass-ceramic and ZnO-enriched glass, respectively, showed macrophages in periapical tissues.

##### Fibrosis

Fibrosis is the end result of chronic inflammation and repair; therefore, we must also consider fibrosis and inflammation. A fibrosis healing pattern was present in 56.6% of the G3T glass-ceramic and 40.0% of the ZnO-enriched glass study groups. These values were very similar to the positive control group (57.1%). The presence of collagen fibrosis was lower in the root canal samples sealed with G3T glass-ceramic (11.1%) than those sealed with ZnO-enriched glass (40%) (Figure 2).

##### Vascularization

**Figure 3:**
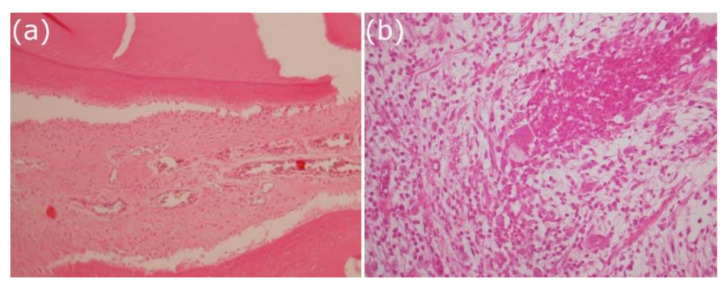
Representative images (×400) of blood vessel neoformation: (**a**) Associated with the G3T glass-ceramic study group (lower density); (**b**) associated with the ZnO-enriched glass study group (higher density).

The G3T glass-ceramic (44.4%) and ZnO-enriched glass (50.0%) both evidenced blood vessel neoformation in the periapical region (Figure 3).

### 2.3. Microbiological Results

Four microbiological samples were collected during the clinical procedures. The first sample, labeled the “control culture sample”, was collected as a negative control to assess the sterility of the dental crown and rubber dam. After making an access cavity, the root canal system was performed and remained open for one week. Then, the access cavity was temporary sealed with temporary cement to induce bacterial contamination and to promote apical periodontitis. Two weeks later, another microbiological sample was collected (the “baseline sample”), after irrigating the root canal system with a sterile saline solution. Afterwards, the root canal system was cleaned and shaped with an R25 rotary file. The root canal system was irrigated with NaOCl, ethylenediaminetetraacetic acid (EDTA), and a sterile saline solution, using an endodontic needle. Then, NaOCl was deactivated with 5% Na_2_S_2_O_3_, and the root canal system was dried with sterile paper points, so that the third microbiological sample was obtained (the “root canal treatment sample”). After submitting the animals to the euthanasia process, additional microbiological samples were obtained “the postmortem sample”, to analyze the residual bacteria present in the root canal system and dentinal tubules, which had not been detected in the previous samples.

**Figure 4:**
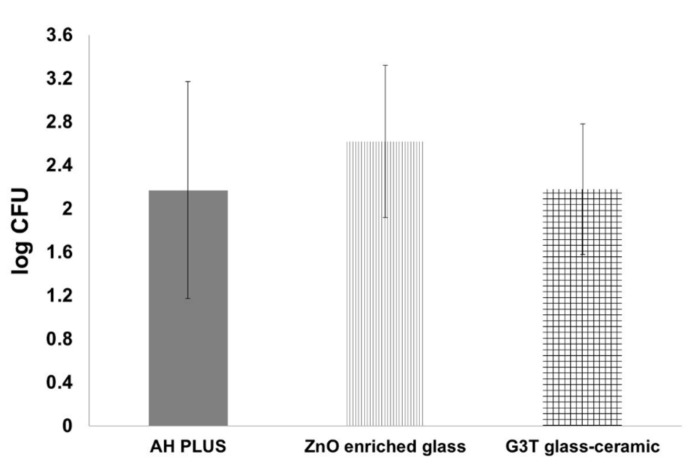
Microbiological values (log CFU) of the two-week contamination period (“baseline sample”) sample and the study groups. Bars represent standard error.

Cultivable bacteria were not detected in the “control culture sample”. The microbiological results of the two-week contamination period (“baseline sample”) sample (Figure 4) showed non-significant bacterial loads (*p* = 0.264) between the G3T glass-ceramic study group (151 ± 186.6 colony forming units (CFU)), the ZnO-enriched glass study group (415.8 ± 717.2 CFU), and the positive control group (146.7 ± 210.0 CFU).

Likewise, no significant differences were found between the total bacterial reduction values in the “root canal treatment sample” and the “postmortem sample” (data not shown). These results provide evidence of the efficacy of the chemical mechanical clinical procedure; however, the procedure prevented the analysis of the bactericidal capacity of the additives incorporated into the epoxy resin-based sealer, due to their absence in the periapical region.

### 2.4. Radiological Results

**Figure 5:**
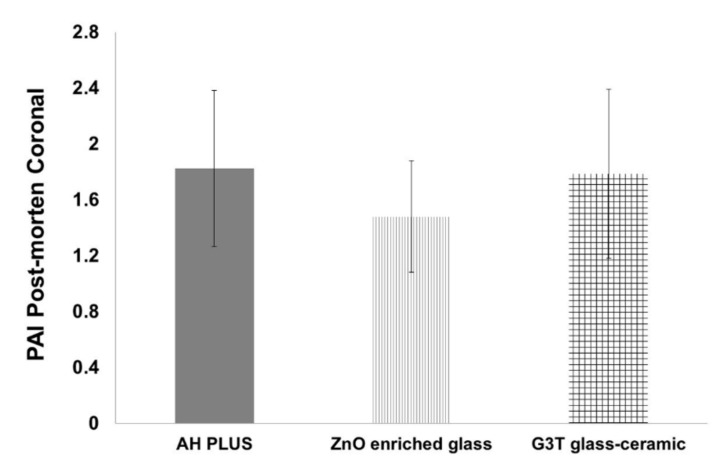
Relation between additive agents and postmortem PAI values. Plots represent mean values with 95% confidence intervals. Bars represent standard error.

The periapical index (PAI) is a scoring system for evaluating apical periodontitis via radiographs, which uses a scale from one to five, ranging from healthy to severe periodontitis with exacerbated features, respectively [20]. We observed no significant radiographic reduction in the postmortem PAI values between the AH Plus group and the G3T glass-ceramic (*p* = 0.885) or ZnO-enriched glass (*p* = 0.169) groups, in the coronal plane after 2 months healing (Figure 5). The highest reduction was observed in the sagittal plane of the G3T glass-ceramic study group.

### 2.5. Field Emission Scanning Electron Microscopy (FE-SEM) with Energy Dispersive X-ray Expectroscopy (EDS)

**Figure 6:**
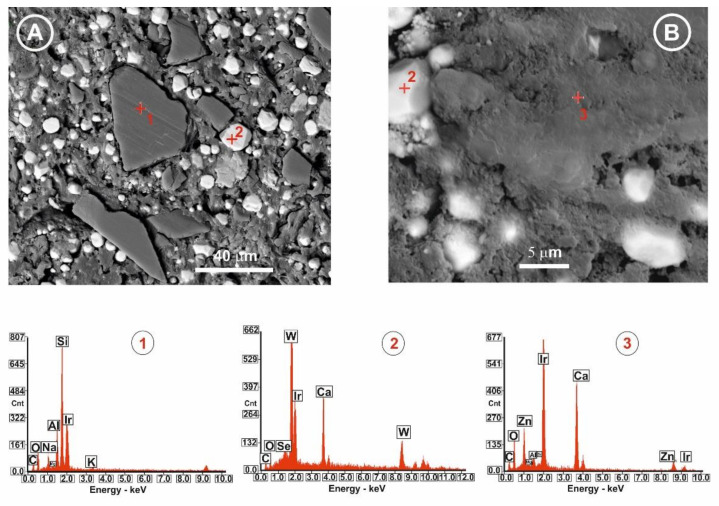
(**A**,**B**) Backscattered FE-SEM micrographs; (**1**,**2**,**3**) EDS microanalysis, of the selected spots (red cross with the corresponding numbers) of the dental sealer containing the bactericidal materials, (**A**) G3T glass-ceramic and (**B**) ZnO-enriched glass, both located at the delta apical of the canal.

Figure 6 shows the microstructure and chemical analysis of the apical section of the tooth after being cut and polished. The results demonstrate the homogeneous incorporation of both antimicrobial materials into the epoxy-amine matrix. One component of AH Plus, according to the manufacturer, is calcium tungstate particles which are clearly identified by FE-SEM (average particle size of 8 µm) in both types of samples (Figure 6), and EDS analysis also corroborates their chemical composition. G3T glass-ceramic is composed of combeite (Na_4_Ca_4_(Si_6_O_18_)) and nepheline (Na_6.65_Al_6.24_Si_9.76_O_32_) dispersed in a glassy matrix. Crystals of nepheline are clearly visible in the resin containing the glass-ceramic (Figure 6A). The ZnO-enriched glass particles in the resin appear to be slightly agglomerated (Figure 6B).

## 3. Discussion

In this study, we evaluate and compare the antibacterial effect and tissue response of two bioactive glass-based additives incorporated in a commercial epoxy resin-based sealer.

The bactericidal effectiveness of the glass-filled endodontic sealer was evaluated against *E. faecalis*, the most commonly used microorganism in in vitro studies relevant to persistent periapical infections. The in vitro tests carried out to investigate the antibacterial activity of the glass-filled AH Plus during the sealer’s setting process (Figure 1) have shown that the bactericidal activity of AH Plus was time limited and was no longer detectable after 48 h of mixing (Figure 1c). These results are in accordance with those of other previous studies [10,21,22,23]. The antibacterial activity of AH Plus sealer remains through its setting process, and it is mediated either by the amines released during setting or by the bisphenol A diglycidyl ether [21,24]. One disadvantage of the loss of the antibacterial components of AH Plus is that it leads to the shrinkage of the sealer which can endanger the sealing effect [25].

The sealer with 15 wt% glass-ceramic retained its bactericidal effect throughout the experimental time period (Figure 1). The development of a root canal sealer with long-term antibacterial activity is a challenge. Current commercial endodontic sealers have short-term antibacterial activities, which are attributed to the release of the antibacterial ingredients in the sealers prior to their fixation; once these bactericidal components are released their antimicrobial activities are lost [26]. The antimicrobial materials (G3T glass-ceramic and ZnO-enriched glass) are both highly durable. They act as a delivery system that can provide sustained release of the ions. ZnO-enriched glass is an excellent dispenser of Zn^2+^ ions to the media for a very long period of time, in the range of years.

Nevertheless, G3T glass-ceramic needs a close contact with the target cells, and acts by membrane depolarization of the microorganisms attached to the glass surface [18,19].

Bioactive glass 45S5 is known to possess antimicrobial activity [27] and the ability to remineralize tissues [28]. Khvostenko, D. et al. demonstrated that a 15 wt% addition of this glass to a resin-based dental composite could significantly reduce the extent of bacteria biofilm penetration into pre-existing marginal gaps [29]. In this study, it was proven that the antimicrobial capability of this glass was lost 7 days after mixing; only the sealer with the glass-ceramic maintained its bactericidal capability (Figure 1). The antimicrobial activity of root canal sealer with G3T glass-ceramic could help to eliminate residual microorganisms unaffected by chemomechanical preparation of the root canal system, such as in the case of *E. faecalis*, therefore, improving the success rate of endodontic treatment. This is probably a very transcendental result as it allows clinicians to avoid the very negative presence of *E. faecalis* that can induce reinfection of root canals.

From the in vivo results, the data obtained in the G3T glass-ceramic group showed a high reduction in the postmortem PAI (Figure 5) values, although 11.1% of the teeth developed mild inflammation and an inflammatory abscess (Figure 2). The presence of low values of macrophages in both study groups indicates acute inflammation of short evolution, but its persistence over time could evidence infection and delayed healing. High lymphocytes levels present in both study groups, indicate evolution to the chronicity of the process, but over time, they are replaced by a fibrosis process. The presence of fully developed newly formed blood vessels indicates a chronic evolution of the process and explains the inflammatory infiltrate present in all groups. This healing pattern can be due to the absence of direct contact between the bactericidal additive and periapical tissues and the absence of patency during the root canal treatment. The impossibility to maintain apical patency, due to the apical anatomy of dog’s teeth, could produce an accumulation of dentinal remnants in the apical region that could cause blockage of the principal root canal, avoiding the effect of sodium hypochlorite and the antimicrobial materials [30]. Previous studies have reported that the antibacterial effect of glass enriched with CaO (G3T) relied on close contact with the bacterial cells [31,32]. Nevertheless, the antibacterial action of the ZnO-enriched glass is attributed to the release of Zn^2+^ ions, which has been proven to be effective as an abutment coating to prevent peri-implant diseases and to inhibit anaerobic bacterial growth [33]. Anaerobic species are highly prevalent in persistent endodontic infections and *E. faecalis* has been reported to be the most predominant bacterial biotype in secondary endodontic infections, being present between 10–76% of persistent endodontic infections after root canal therapy [34]. The difference between these results and those of our previous study [33] is that the coated abutment surface remains in direct contact with the experimental peri-implantitis biofilm; nevertheless, NaOCl can penetrate to a depth of 130 µm into the dentinal tubules [35], while *E. faecalis* is able to penetrate up to a depth of 151 μm and to adhere to collagen in the presence of human serum [36], leaving bacteria harbored in deeper layers, accessory canals, anastomoses, and fins [25].

The sealer penetration into dentinal tubules is considered to be clinically relevant for preventing bacterial recolonization, and allows bacterial inactivation as a blocking agent that entombs residual bacteria within dentinal tubules. Nevertheless, the sealer penetration decreases from the coronal to the apical level, because of a reduction in the diameter and number of dentinal tubules and the presence of dentinal sclerosis [37]. Therefore, the addition of a bactericidal compound is required to avoid the micro leakage over time [38].

As can be seen in Figure 6, through the followed process (a gutta-percha system and an AH Plus epoxy-amine resin-based cement containing one of the studied bactericidal glass-based additives) a perfect distribution of the particles of both glasses has been achieved in the cement located at the delta apical of the canal. In this way, it has been possible to improve the functionality of the dental sealer and, in turn, to locate glass particles with a high bactericidal activity at the apical vertex of the teeth.

Overall, it can be seen that in the cases of dental sealer containing ZnO-enriched glass, scarring is observed with the formation of fibrous tissue. Some cases which did not develop an abscess, showed a good resolution rate with mature collagenous fibrosis (40%). This fact indicates that Zn^2+^ ions spread and diffuse beyond the apex, and then the size of the abscess begins to decrease. In the case of the dental sealer containing G3T glass-ceramic, the bactericidal action was by contact [19,31,32], and therefore this effect was not produced to the same extent as the dental sealer containing ZnO-enriched glass.

Following a conventional root canal treatment (RCT) procedure, it has been possible to place the dental sealer AH Plus containing the different bactericidal glass and glass-ceramic in an apical position without any type of inconvenience caused by a possible change in the rheology of the AH Plus original cement, that is to say, the endodontists who performed the conventional RCT procedure in the in vivo tests observed no appreciable difference in the manipulation of the gutta-percha when the mixture was used. In the literature, it has been well reported that composites containing up to 15 wt% by weight non-silanated bioactive glass filler can have mechanical properties comparable to, or superior to, commercial composites and that the addition of bioactive glass fillers does not endanger the degree of monomer conversions [39].

Biocompatibility is one of the main properties of root canal sealers, as these materials come into direct contact with periradicular tissues [17]. Ensuring that bactericidal additives are compatible with mammalian cells is of paramount importance to allow their placement inside human teeth. In this sense, it is important to point out that both the glass and glass-ceramic used in this study are biocompatible. Evaluations of the cytotoxicity of these materials have been carried out in previous studies through in vitro [18,19] and in vivo tests [33,40].

Apart from the antimicrobial properties and biocompatibility, an ideal root canal sealer should also induce the formation of mineralized tissue. The histological response of the glass-ceramic G3T in the form of a series of rods implanted in the jaws of Beagle dogs was previously studied for a four-month period [40]. Direct growth and bone formation at the rod implant surface was observed. This newly formed bone underwent normal growth. After four months, the average amount of new immature bone, plus the apparent dissolved/attacked rod layer in the implanted rods, was found to be 70 ± 14 vol% of the total volume of the rod. Further investigation is required to more precisely delineate and validate a possible improvement of the osteogenic properties of the sealer with this glass-ceramic.

## 4. Materials and Methods

### 4.1. In Vitro Evaluation of the Bactericidal Effect against E. faecalis

Three types of antimicrobial glasses were incorporated in a commercial epoxy-amine resin endodontic sealer (AH Plus) used for root canal treatment. AH Plus was mixed with 0 and 15 wt% of each glass, handled in accordance with the manufacturers´ recommendations.

The selected antimicrobial glasses were: (i) an antimicrobial glass (labeled as ZnO-enriched glass) with the chemical composition (wt%) of 18.7 SiO_2_, 33.2 B_2_O_3_, 5.38 Na_2_O, 34.7 ZnO, 4.97 Al_2_O_3_, 2.25 ZrO_2_, and 0.8 others, and with a particle size distribution < 20 µm (d_50_ 7 µm and d_90_ 13 µm) (Nanoker S.L., Oviedo, Spain); (ii) an antimicrobial glass-ceramic powder (labeled as G3T glass-ceramic) with the chemical composition (wt%) of 41.6 SiO_2_, 20.0 Na_2_O, 19.5 CaO, 10.1 Al_2_O_3_, 6.4 B_2_O_3_, 0.21 MgO, 0.11 Fe_2_O_3_, 0.23 ZrO_2_, 0.14 TiO_2_, 0.61 K_2_O, and 1.1 others, and with a particle size distribution < 45 µm (d_50_ 10 µm and d_90_ 37 µm) (Nanoker S.L., Oviedo, Spain), and (iii) a commercial bioglass (45S5 Bioglass^®^, Novabone Products LLC, Alachua, FL, USA) with the chemical composition (wt%) of 45 SiO_2_, 24.5 Na_2_O, 24.5 CaO, and 6 P_2_O_5_, and with a particle size distribution < 20 µm (d_50_ 7 µm and d_90_: 17 µm).

The test was performed in a 48-well flat bottom microtiter plate. The bottoms of the wells were carefully coated with the sealer containing each antimicrobial glass (3 wells for each tested material). The bactericidal activity of these glass-filled sealers were tested under different conditions: (1) samples were used within 20 min after mixing, (2) samples set for 48 h in a humid atmosphere at 37 °C, (3) samples set for 48 h in a humid atmosphere at 37 °C, and then were aged for 5 days in phosphate buffered saline (PBS) at 37 °C.

Several in vitro studies have investigated the antimicrobial activity of root canal sealers by using various methods. The most commonly used methods include agar diffusion tests (ADTs) and direct contact tests (DCTs) [26]. In this study, a DCT was performed to evaluate the antimicrobial activity of the glass-filled sealers developed. The DCT was based on measuring the effect of close contact between test bacteria and the endodontic sealers on the kinetics of bacterial outgrowth. This method mimics the direct contact between microorganisms and the endodontic sealers inside a root canal [41]. *E. faecalis* was used as the test bacterium. It is the most resistant species to eliminate from the root canal and can survive in root-filled canals even without the support of other bacteria and with scant substrate [21,26]. After mixing, a 10 µL suspension (ca. 10^6^ bacteria) of a standard strain of *E. faecalis* (ATCC 29212) was placed on the test material for 20 min, 24 h, or 7 days. The bacteria were allowed to directly contact the sealers for 1 h at 37 °C in a humid atmosphere. Brain Hearth Infusion (BHI) (Scharlab S.L., Barcelona, Spain) broth (1 mL) was added to each well and gently mixed for 2 min. Then, the bacterial growth in the presence of the glass-filled sealer was measured at different times (after 3, 6, and 24 h) by serial dilution plating.

### 4.2. In Vivo Procedures

#### 4.2.1. Study Design

According to the study conducted by Stambolsky et al. [42], thirty single-rooted teeth from five male Beagle dogs (four years old) were selected at random. A controlled clinical trial was conducted in accordance with the ethical principles of the ARRIVE guidelines and were carried out in accordance with the UK Animals (Scientific Procedures) Act 1986, the associated guidelines, and EU Directive 2010/63/EU for animal experiments. The study protocol was approved by the Ethics Committee for Animal Research Welfare to be carried out at the Minimally Invasive Surgery Center, in Caceres (Spain), between May and August 2017.

#### 4.2.2. Clinical Procedure

**Figure 7:**
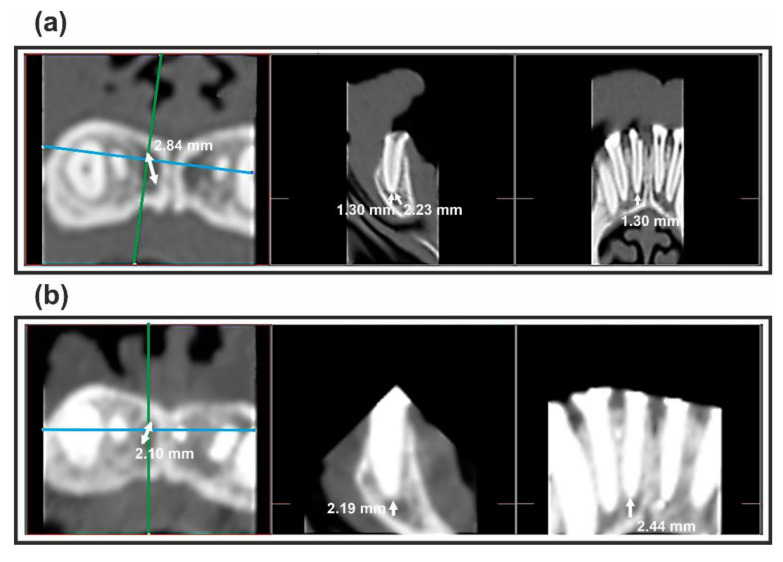
Measurements of the apical lesion of one of the teeth on the axial, sagittal, and transverse planes: (**a**) The pre-mortem computerized axial tomography (CAT) and (**b**) the postmortem cone beam computed tomography (CBCT), through the 3D implant planning software.

Veterinary assistance was given throughout the study. General anesthesia was induced with intravenously injected propofol 10 mg/kg (Propofol Hospira, Hospira Productos Farmacéuticos y Hospitalarios, Madrid, Spain) and sustained with sevofluorano (Sevorane, Abbott Laboratories, Madrid, Spain). One No. 7 endotracheal tube with a balloon cuff was placed and connected to a circular anesthesia circuit (Leon Plus, Heinen & Löwenstein, Bad Ems, Germany). Multimodal analgesia was used in the perioperatory care, employing ketorolac 1 mg/kg (Toradol 30 mg, Roche Farma S.A., Madrid, Spain), tramadol 1.7 mg/kg (Grünenthal GmbH, Aachen, Germany), and buprenorfine 0.01 mg/kg (Buprex, Reckitt Benckiser Pharmaceuticals Limited, Berkshire, UK). The rubber dam (Hygenic^®^ dental dam, Coltene^®^ Whaldent Gruppe, Altstätten, Switzerland) was disinfected with a povidone iodine solution (Betadine^®^, MEDA, Solna, Sweden). The crown and rubber dam were disinfected with 5 mL of 30% hydrogen peroxide (Cinfa, Navarra, Spain) for 30 s and 5 mL of 2.5% sodium hypochlorite (NaOCl) (Clorox, Oakland, CA, USA) for another 30 s. NaOCl deactivation was achieved by irrigation with 5 mL of 5% sodium thiosulfate (Na_2_S_2_O_3_) for 30 s [43]. A microbiological sample was collected as a negative control, to assess the sterility of the dental crown and rubber dam (“control culture sample”). After making the access cavity, the working length was established by an electronic apex locator (Root ZX^®^, Morita^®^, Tokio, Japan). Then, the root canal system was performed by Hëdstrom^®^ files up to a size 20/0.02 (Colorinox, Dentsply Sirona^®^, Ballaigues, Switzerland), and remained open for one week. Then, the access cavity was temporarily sealed with temporary cement (Cavit™, 3M ESPE, Saint Paul, MN, USA) to induce bacterial contamination and promote apical periodontitis [44]. Two weeks later, a pre-mortem computerized axial tomography (CAT) (Philips CT Brillance 6 Slices, Philips Medical Systems, Philips Medical Systems, Eindhoven, The Netherlands) was performed to confirm apical lesion (Figure 7a).

After that, root canal system was irrigated with 1 mL of sterile saline solution (Braun^®^, Jaen, Spain). Afterwards, another microbiological sample was collected (“Baseline sample”), by inserting three sterile paper points (Dentsply Sirona^®^, Ballaigues, Switzerland) into the root canal system for one minute. Next, the samples were transferred into a sterile Eppendorf pellet with 1 mL culture medium (Nutrient Broth^®^, Difco, Detroit, MI, USA). Subsequently, the root canal system was cleaned and shaped with R25 rotary file (Reciproc^®^, VDW, Munich, Germany). The root canal system was irrigated with 5 mL of 5.25% NaOCl, 5 mL of 17% ethylenediaminetetraacetic acid (EDTA) (Smear Clear^®^, SybronEndo, CA, USA) and 5 mL of sterile saline solution; using an endodontic needle (Miraject^®^ Endo Luer, Hager&Werken, Duisburg, Germany). The contact between the irrigating solution and the surface of the root canal walls was enhanced by using a sonic device (Endoactivator^®^, Dentsply Sirona^®^, Ballaigues, Switzerland). Then, NaOCl was deactivated with 5% Na_2_S_2_O_3_, and the root canal system was dried with sterile paper points, so that the third microbiological sample was obtained (“Root Canal Treatment sample”). The root canal system was sealed by a sterile warm gutta-percha system (GuttaCore^®^, Dentsply Sirona^®^, Ballaigues, Switzerland) and an epoxy-amine resin-based cement (AH Plus, Dentsply DeTrey, Konstanz, Germany), containing 15 wt% of one of the two antimicrobial additives used in the in vitro test at random (Epidat 3.1, OPS-OMS, A Coruña, Spain): G3T glass-ceramic (n = 10) and ZnO-enriched glass (n = 10). Both glass powders were subjected to gamma radiation before to be used. Ten teeth (n = 10) were selected as random as positive control group without any bactericidal additive.

The access cavities were filled with a composite restoration (Filtek™ Z250, 3M ESPE, Saint Paul, MN, USA).

#### 4.2.3. Euthanasia Procedure

Animals were randomly euthanized by an overdose of potassium chloride (2 mEq/kg), under a premedication with dexmedetomidine (5 μg/kg) intravenously, followed by an overdose of propofol (15 mg/kg) intravenously.

### 4.3. Histological Analysis

Jaws were fixed in 10% buffered formalin solution for 48 h to allow histopathological processing at the Surgical Pathology Department of San Carlos Clinical Hospital. Then, teeth were extracted and bone fragments were sectioned with an oscillating autopsy saw (Exakt, Kulzer, Germany) in sagittal 5-mm-thick serial sections, decalcified in nitric acid, fixed in crescent alcoholic solutions up to xilol to be embedded in paraffin and after sectioning in 4 micron slides, stained with hematoxylin and eosin (Papanicolau, Merck, Germany) and Masson trichrome staining techniques, and examined under light microscopy (Optiphot 2-POL, Nikon, Tokyo, Japan) by an experienced pathologist blinded to the kind of additive employed in each case.

### 4.4. Microbiological Procedure

Sterile paper points were transferred to a sterile Eppendorf containing 1 mL culture medium and immediately frozen at −20 °C. All microbiological samples were immediately processed; attending to Moller´s method [45], after slowly defrosting at 4 °C for 24 h and vortexing for 5 min. Serial dilutions were prepared, and 100 µL aliquots from each tube were seeded onto blood agar plates (Columbia Agar with 5% sheep blood, 770418 Dismalab SL, Madrid, Spain) and incubated aerobically in a stove at 37 °C for 48 h for CFU determination.

### 4.5. Radiological Assessments

After the animals were sacrificed, a postmortem cone beam computed tomography (CBCT) (Figure 7b) (NewTom GIANO, Verona, Italy) was performed to compare the healing process of the periapical tissues with the pre-mortem computerized axial tomography (CAT) (Figure 7a), measured by means of periapical index (PAI) [20], through a 3D implant planning software (Nemotec Dental Studio^®^, Nemotec S.L., Madrid, Spain).

### 4.6. FE-SEM Examination

**Figure 8:**
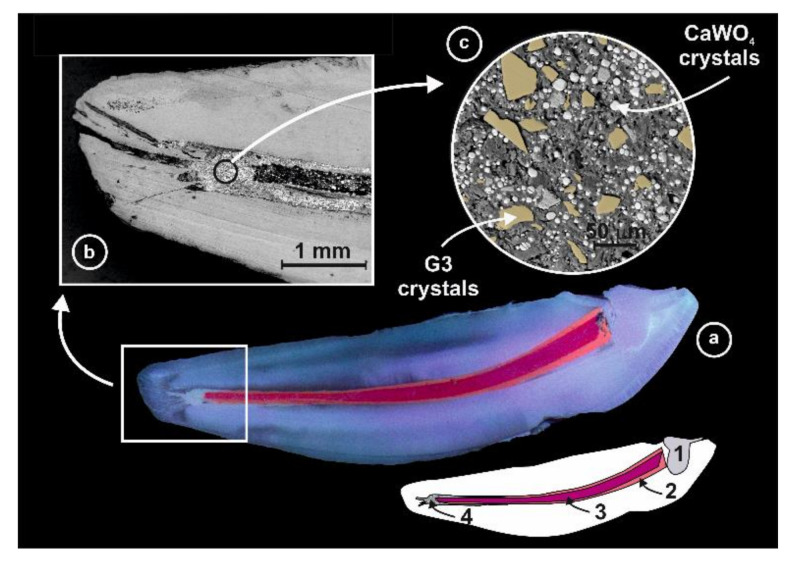
(**a**) Image and schematic view of the sagittal plane of one of the teeth after being polished: (1) composite restoration used to fill the access cavity, (2) gutta-percha, (3) Guttacore, and (4) AH Plus dental sealer containing the bactericidal G3T glass-ceramic located at the apical position. (**b**,**c**) field emission scanning electron micrographs at different magnifications of the apical section.

The distribution pattern of the biocidal particles, present in the epoxy-amine resin matrix, was evaluated by scanning electron microscopy (FE-SEM, FEI Nova NANOSEM 230), after cutting off the teeth in the sagittal plane (Micromet M, Remet, Italy) and subsequent polishing them down 1 µm. A schematic view of the clinical procedure and characterization by FE-SEM it is shown in Figure 8.

### 4.7. Statistical Analysis

All variables of interest were recorded for statistical analysis with SPSS 22.00 for Windows^®^. Descriptive statistics were expressed as means and standard deviation for quantitative variables and as absolute numbers and percentages for qualitative variables. A comparative analysis was performed by comparing the mean colony-forming unit count for each group before and after the intervention using a Mann–Whitney U test, since variables did not have a normal distribution. A *p* ≤ 0.05 was considered to be statistically significant.

## 5. Conclusions

In vitro studies have demonstrated that G3T glass-ceramic showed a longer antibacterial activity, particularly versus *E. faecalis*, than ZnO-enriched glass sealer, and even longer than in the case of a commercial antimicrobial bioactive glass. G3T glass-ceramic and ZnO glass incorporated as additive into a root canal sealer demonstrated a promising antimicrobial effect to avoid apical periodontitis, respecting the integrity of the periapical tissues. Nevertheless, further research is required to ensure direct contact between the biocidal additives and the periapical tissues.

## Data Availability

Data is contained within the article.
